# Further improvement of circuit survival in citrate based continuous renal replacement therapy

**DOI:** 10.1093/ckj/sfae187

**Published:** 2024-06-26

**Authors:** Alena Post, Èmese R H Heijkoop, Lotte L M Diebels, Adrian Post, Matijs van Meurs, Peter H J van der Voort, Casper F M Franssen, Meint Volbeda

**Affiliations:** Department of Critical Care, University of Groningen, University Medical Center Groningen, Groningen, The Netherlands; Department of Critical Care, University of Groningen, University Medical Center Groningen, Groningen, The Netherlands; Department of Critical Care, University of Groningen, University Medical Center Groningen, Groningen, The Netherlands; Department of Nephrology, University of Groningen, University Medical Center Groningen, Groningen, The Netherlands; Department of Critical Care, University of Groningen, University Medical Center Groningen, Groningen, The Netherlands; Department of Critical Care, University of Groningen, University Medical Center Groningen, Groningen, The Netherlands; Department of Nephrology, University of Groningen, University Medical Center Groningen, Groningen, The Netherlands; Department of Critical Care, University of Groningen, University Medical Center Groningen, Groningen, The Netherlands

**Keywords:** acute kidney injury, continuous renal replacement therapy, continuous veno-venous hemodiafiltration, continuous veno-venous hemofiltration, regional citrate anticoagulation

## Abstract

**Background:**

Continuous renal replacement therapy (CRRT) is the most frequently used modality of renal replacement therapy (RRT) in critical care patients with acute kidney injury (AKI). Adequate CRRT delivery can be challenging, due to problems with circuit patency. To improve circuit patency, we developed a new CRRT protocol using continuous veno-venous hemodiafiltration (CVVHDF) with 3.0 mmol/l regional citrate anticoagulation (CVVHDF/RCA3.0) as our first choice RRT modality.

**Methods:**

Retrospective comparison of efficacy and safety of a CVVHDF/RCA3.0 protocol with our former continuous veno-venous hemofiltration protocol with 2.2 regional citrate anticoagulation (CVVH/RCA2.2) in adult critically ill patients with AKI requiring CRRT between 25 April 2020 and 24 October 2021.

**Results:**

In total, 56 patients (257 circuits) and 66 patients (290 circuits) were included in the CVVH/RCA2.2 and CVVHDF/RCA3.0 groups, respectively. Median circuit survival was significantly higher in patients treated with CVVHDF/RCA3.0 (39.6 (IQR 19.5–67.3) hours) compared to patients treated with CVVH/RCA2.2 (22.9 (IQR 11.3–48.6) hours) (*P *< .001). Higher body weight and higher convective flow were associated with a lower circuit survival. Metabolic control was similar, except for metabolic alkalosis that occurred less frequently during CVVHDF/RCA3.0 (19% of patients) compared to CVVH/RCA2.2 (46% of patients) (*P *= .006).

**Conclusions:**

CRRT circuit survival was longer with CVVHDF/RCA3.0 compared to CVVH/RCA2.2. CRRT circuit survival was negatively associated with higher body weight and higher convective flow.

KEY LEARNING POINTS **What was known:**

- Continuous renal replacement therapy is the most frequently used modality of renal replacement therapy in critical care patients with acute kidney injury.
- Continuous renal replacement therapy circuit survival is longer with regional citrate anticoagulation compared to heparin.
- Adequate continuous renal replacement therapy delivery can be challenging due to problems with circuit patency.

**This study adds:**

- Continuous renal replacement therapy circuit survival time was significantly longer with continuous veno-venous hemodiafiltration with 3.0 mmol/l regional citrate anticoagulation and routine heparin priming compared to continuous veno-venous hemofiltration with 2.2 mmol/l regional citrate anticoagulation without routine heparin priming.
- Higher body weight and higher convective flow were associated with a lower circuit survival.
- Metabolic alkalosis was less frequent with continuous veno-venous hemodiafiltration with 3.0 mmol/l regional citrate anticoagulation compared to continuous veno-venous hemofiltration with 2.2 mmol/l regional citrate anticoagulation, due to a lower bicarbonate concentration in the post-filter substitution and dialysate fluid (22 versus 30 mmol/l).

**Potential impact:**

- The performance of continuous renal replacement therapy protocols with regional citrate anticoagulation (RCA) will improve with a change from continuous veno-venous hemofiltration with RCA 2.2 mmol/l to continuous veno-venous hemodiafiltration with RCA 3.0.

## INTRODUCTION

Continuous renal replacement therapy (CRRT) is the most frequently used modality of renal replacement therapy (RRT) in critical care patients with acute kidney injury (AKI) [1]. The debate regarding CRRT dose prescription in patients with AKI has been settled with guidelines advising a dose of 20-25 ml/kg/h [2, 3]. The actual delivery of this dose, however, can be challenging due to clotting in the CRRT circuit. Especially in patients with obesity and in patients with COVID-19 there is an increased risk for premature CRRT circuit failure [4]. After circuit failure, the circuit needs to be replaced, which leads to temporal discontinuation (downtime) of treatment. To increase the CRRT circuit patency and dose delivery, we developed a new CRRT protocol with continuous veno-venous hemodiafiltration (CVVHDF) with 3.0 mmol/l regional citrate anticoagulation (RCA) (CVVHDF/RCA3.0) as our first choice RRT modality.

The aim of this study is to give a detailed analysis of the efficacy, safety, and practical implication of the CVVHDF/RCA3.0 protocol compared to our former CRRT protocol with continuous veno-venous hemofiltration (CVVH) with 2.2 mmol/l RCA (CVVH/RCA2.2).

## MATERIALS AND METHODS

### Design

This retrospective observational cohort study was performed in the ICU of the University Medical Center Groningen (UMCG), The Netherlands and was conducted according to the principles of the Helsinki declaration. The local Medical Ethics Review Board reviewed and waived (M22.289857) this study.

### Population

All adult critically ill patients with (acute) kidney injury requiring CRRT between 25 April 2020 and 24 October 2021.

### CRRT treatment protocols

The decision to start CRRT was made by the treating ICU physician in consultation with the nephrologist. CRRT was performed using the Prismax^®^ (Baxter, Brooklyn Park, MN, USA) with Prismaflex ST 150^®^ filterset (Baxter, Meyzieu Cedex, France). Venous access was obtained with a double-lumen 13F central venous catheter (high-flow double-lumen catheter; Baxter, Hechingen, Germany).

Comprehensive descriptions of the CVVH/RCA2.2 and CVVHDF/RCA3.0 protocols are given in Appendix A.

### Patients and allocation to treatment groups

All patients treated with CRRT between 25 April 2020 and 24 January 2021 (period 1) were treated according the CVVH/RCA2.2 protocol and are referred to as the “CVVH/RCA2.2 group.” All patients treated with CRRT between 25 January 2021 and 24 October 2021 (period 2) were treated according to the CVVHDF/RCA3.0 protocol and are referred to as the “CVVHDF/RCA3.0 group.”

### Data collection

Patient and treatment data were routinely and prospectively stored in the UMCG standardized electronic health record system (EPIC Systems, Verona, WI, USA). Data were entered in REDCap (Research Electronic Data Capture hosted at the UMCG). Collected data included: [1]. patient characteristics [age, sex at birth, admission diagnosis, the Acute Physiology and Chronic Health Evaluation score 4 (APACHE IV) score, presence of COVID-19 and chronic kidney disease (CKD), indication for CRRT, body length, and weight, type, and dose of anticoagulants], [2]. Daily laboratory data recorded were: hematocrit, PT, aPTT, fibrinogen, total calcium, albumin, phosphate, and magnesium levels, and multiple times per day point of care blood-gas analyses including measurements of pH, pCO_2_, HCO_3_^−^, chloride, lactate, iCa, sodium, and potassium, [3]. Detailed CRRT treatment data taken were: blood flow (ml/min), convective flow (pre- and post-filter substitution flows) (ml/hour), citrate dose (mmol/l), dialysate flow (ml/hour), net fluid removal (ml/hour), ultrafiltration rate (daily minimum and maximum values in ml/hour), location and length of double-lumen central venous catheter, and CRRT circuit survival time (hours).

### Primary outcome

The primary outcome was CRRT circuit survival time in hours. For this primary outcome analysis, we excluded circuits that were terminated because of [1] diagnostic procedures or interventions (operations) outside the ICU [2], technical errors of the Prismax, and [3] circuits that were ended because of the decision to cease CRRT, since the survival time of these circuits does not reflect the performance of the CRRT protocols.

### Secondary outcomes

Secondary outcomes were: [1] delivered CRRT dose expressed as mean delivered effluent dose in ml/kg/hour during the full CRRT treatment duration [2]; proportion of patients who developed: (i) a clinically relevant metabolic alkalosis, defined as pH > 7.45 and HCO_3_^−^ >28 mmol/l; (ii) non-lactate high anion gap metabolic acidosis [(Na–Cl–HCO_3_^−^–lactate) + 0.25 (40–serum albumin)] >12 mmol/l); (iii) increased total Ca/iCa ratio (>2.25); (iv) hypophosphatemia (<0.7 mmol/l); and (v) sodium derangements (Na < 135 and >145 mmol/l) [3] and recovery of metabolic acidosis (pH ≥ 7.38 and HCO_3_^−^ ≥22) [5]. For these analyses, we included only patients who underwent complete per protocol treatment.

### Sensitivity analyses

Since COVID-19 may influence the circuit survival time [4, 6, 7], we analyzed the circuit survival separately for patients with and without COVID-19. To facilitate a comparison with other studies, we also analyzed the median circuit survival time of the first circuit only. We also analyzed median survival time of all circuits, without exclusion of circuits that were terminated because of procedures outside the ICU, technical errors, or because of the decision to cease CRRT. The incidence of metabolic alkalosis was also analyzed according to a more sensitive definition: pH > 7.38 and HCO_3_^−^ >26 mmol/l [4] in patients who underwent a complete per protocol treatment.

### Statistical analysis

Data were analyzed with R version 4.2.2 (Vienna, Austria) (http://cran.r-project.org/). Baseline data are expressed as means (± SD) 95% or medians (interquartile ranges). Comparisons between the groups were made using Student's *t*-test, chi square (two sided), or Mann–Whitney *U*-test, as appropriate. *P *< .05 was considered statistically significant. CRRT circuit survival was analyzed using frailty Cox proportional hazard models, adjusted for sex, body weight, age, COVID-19, and APACHE IV score.

## RESULTS

**Figure 1:**
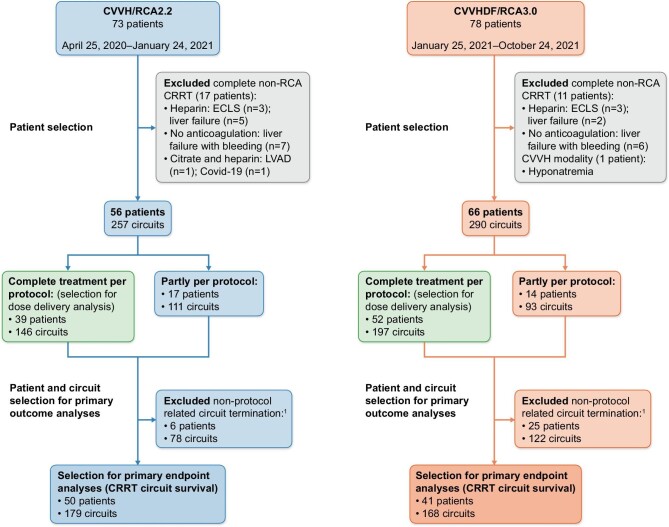
Flowcharts of patients and circuit selection in both protocol groups. ^1^ = circuit termination because of (i) diagnostic procedures or interventions outside the ICU, (ii) technical errors of the Prismax, and (iii) circuits that were ended because of the decision to cease CRRT. Reasons for partial non-protocol treatment in period 1 were: heparin: ECLS (extracorporeal life support system) treatment (two patients); frequent clotting (five patients), no anticoagulation: therapeutic anticoagulation due to thrombosis (one patient); liver failure (one patient), citrate, and heparin: COVID-19 (six patients), ECLS (one patient), other: modality switch from study period 1 to period 2 with exclusion of CRRT circuits of period 2 (one patient). Reasons for partly non-protocol treatment in period 2 were: heparin: ECLS (five patients); liver failure (two patients), no anticoagulation: liver failure with bleeding tendency (two patients), citrate and heparin: ECLS (two patients); COVID-19 (three patients).

Between 25 April 2020 and 24 October 2021, 151 patients were treated with CRRT in our ICU (Fig. 1). Seventeen and 12 patients in period 1 (CVVH/RCA2.2) and period 2 (CVVHDF/RCA3.0), respectively, were excluded because they were not treated according to the per protocol RRT modality. This resulted in 56 and 66 patients in the CVVH/RCA2.2 group and the CVVHDF/RCA3.0 group, respectively. In the CVVH/RCA2.2 group, 39 patients (70%) received a complete per protocol treatment, whereas 17 patients did not solely receive CRRT with RCA for several reasons (Fig. 1). In the CVVHDF/RCA3.0 group 52 patients (79%) received a complete per protocol treatment, whereas 14 patients did not solely receive CRRT with RCA (Fig. 1). Median CRRT durations were 3.6 and 4.4 days in the CVVH/RCA2.2 and the CVVHDF/RCA3.0 groups, respectively. The median numbers of circuits per patient were three and two in the CVVH/RCA2.2 and the CVVHDF/RCA3.0 groups, respectively.

### Patient characteristics at CRRT start

Age, sex distribution, APACHE IV score, and the proportion of patients with COVID-19 and CKD were comparable for both groups (Table 1).

**Table 1:** Baseline characteristics at ICU admission.

	CVVH/RCA2.2	CVVHDF/RCA3.0	
	*n* = 56	*n* = 66	*P* value
Age (mean)	58.8	60.0	.71
Female	18 (32%)	21 (32%)	.97
Length (cm) (mean ± SD)	177 (11)	176 (10)	.52
Weight (kg) (mean ± SD)	92.2 (23.0)	91.3 (19.5)	.96
BMI (kg/m^2^) (mean ± SD)	29.3 (5.7)	30.0 (6.3)	.83
Apache IV score (mean ± SD)	78.7 (25.3)	88.5 (27.0)	.08
Chronic kidney insufficiency	11 (20%)	11 (17%)	.67
Admission diagnosis			
Medical patients			
severe sepsis/septic shock	4 (7.1%)	12 (18%)	.07
cardiogenic shock	5 (8.9%)	5 (7.6%)	>.99
respiratory failure	10 (18%)	9 (14%)	.52
Surgical patients			
post-transplantation	8 (14%)	6 (9.1%)	.37
heart transplant	5	2	.28
liver transplant	1	3	.12
kidney transplant	1	0	.37
lung transplant	1	1	.83
post-vascular surgery	4 (7.1%)	3 (4.5%)	.70
post-cardiothoracic surgery	5 (8.9%)	9 (14%)	.42
COVID-19 positive	18 (32%)	15 (23%)	.24
mechanical ventilation at CRRT start	49 (88%)	57 (85%)	.85
vasoactive drugs at CRRT start	52 (93%)	58 (88%)	.36

At ICU admission, 64% of patients in the CVVH/RCA2.2 group received mechanical ventilation compared to 67% of patients in the CVVHDF/RCA3.0 group (*P *= .78). At CRRT start, 88% of patients in the CVVH/RCA2.2 group received mechanical ventilation compared to 85% in the CVVHDF/RCA3.0 group (*P *= .85). At ICU admission, 82% of patients in the CVVH/RCA2.2 group received vasopressors compared to 56% in the CVVHDF/RCA3.0 group (*P *= .002). At CRRT start, 93% of patients in the CVVH/RCA2.2 group received vasopressors compared to 88% in the CVVHDF/RCA3.0 group (*P *= .36) (Table 1). Laboratory parameters prior to CRRT start did not differ significantly between the groups (Table 2).

**Table 2:** Laboratory parameters prior to the start of CRRT.

	CVVH/RCA2.2^a^	CVVHDF/RCA3.0	*P* value
	*n* = 56	*n* = 66	
Creatinine (µmol/l) (mean ± SD)	400.3 (230.8)	363.6 (210.1)	.38
Urea (mmol/l) (mean ± SD)	27.4 (11.7)	28.0 (15.1)	.99
pH (kPa) (mean ± SD)	7.3 (0.1)	7.3 (0.1)	.57
pCO2 (kPa) (mean ± SD)	6.3 (2.5)	5.7 (1.6)	.61
HCO3^−^ (mmol/l) (mean ± SD)	22.1 (6.0)	20.5 (5.9)	.11
Sodium (mmol/l) (mean ± SD)	138.1 (5.9)	138.0 (6.9)	.58
Potassium (mmol/l) (mean ± SD)	5.2 (0.9)	5.2 (0.9)	.63
Aniongap (mmol/l) (mean ± SD)	14.7 (3.9)	13.2 (5.0)	.046
Lactate (mmol/l) (mean ± SD)	1.7 (1.8)	2.6 (2.9)	.1
iCa (mmol/l) (mean ± SD)	1.0 (0.1)	1.1 (0.1)	.32

^a^Data to calculate the anion gap were missing in two patients in the CVVH/RCA2.2 group.

CRRT was most often initiated because of fluid overload, anuria or oliguria (in 55% and 47% of patients in the CVVH/RCA2.2 and CVVHDF/RCA3.0 group, respectively; *P *= .36), followed by hyperkaliemia (in 50% and 41% of patients in the CVVH/RCA2.2 and CVVHDF/RCA3.0 groups, respectively; *P *= .31) and metabolic acidosis (in 13% and 20% of patients in the CVVH/RCA2.2 and CVVHDF/RCA3.0 groups, respectively; *P *= .28) (Table S5, Appendix B).

### Application of CRRT treatment

Overall, 547 per protocol CRRT circuits were used: 257 and 290 circuits in the CVVH/RCA2.2 and the CVVHDF/RCA3.0 group, respectively (Fig. 1). After exclusion of CRRT circuits that were stopped because of procedures outside the ICU, machine errors or the decision to cease CRRT, 179 circuits and 168 circuits in the CVVH/RCA2.2 and the CVVHDF/RCA3.0 groups, respectively, remained for analyses. Double-lumen venous catheters were located in the right jugular vein during 66% and 47% of CRRT circuit runs in the CVVH/RCA2.2 and CVVHDF/RCA3.0 group, respectively (*P *< .001) (Table 3). Ultrafiltration rates did not significantly differ between both groups. Therapeutic dosages of low molecular weight heparins (LMWH) were used in 25% and 23% of patients in the CVVH/RCA2.2 and CVVHDF/RCA3.0 groups, respectively (*P *= .55) (Table 3).

**Table 3:** CRRT circuit characteristics.

	CVVH/RCA2.2	CVVHDF/RCA3.0	
	*n* = 257	*n* = 290	*P* value
Location of central venous catheter			
Right jugular vein	170 (66.1%)	137 (47.2%)	<.001
Left jugular vein	24 (9.3%)	54 (18.6%)	.002
Right femoral vein	37 (14.4%)	59 (20.3%)	.07
Left femoral vein	24 (9.3%)	27 (9.3%)	.99
Right subclavian vein	0 (0%)	6 (2.1%)	.02
Left subclavian vein	0 (0%)	1 (0.3%)	.35
Not applicable due to connection of the CRRT system to ECLS	2 (0.8%)	6 (2.1%)	.21
Prone positioning	30 (11.7%)	33 (11.4%)	.91
Net fluid removal rate (ml/hour)			
0	73 (24.7%)	65 (22.4%)	.11
1–100	81 (31.5%)	84 (29.0%)	.52
101–200	75 (29.2%)	95 (32.8%)	.37
201–300	13 (5.1%)	12 (4.1%)	.61
NA	15 (5.8%)	34 (11.7%)	.02
Anticoagulation			
LMWH—prophylactic dose	177 (69%)	201 (69%)	.95
LMWH—therapeutic dose	65 (25%)	67 (23%)	.55
Vitamin K antagonist	6 (2.3%)	4 (1.4%)	.53
Carbasalate calcium	13 (5.1%)	13 (4.5%)	.75
P2Y12-inhibitor	8 (3.1%)	7 (2.4%)	.62
Enoxaparin	0 (0%)	3 (1.0%)	.25
Unfractionated heparin	0 (0%)	0 (0%)	
DOAC	0 (0%)	0 (0%)	
Laboratory parameters			
Hemoglobin (mmol/l) (median (IQR))	4.9 (4.5–5.4)	4.7 (4.4–5.3)	.006
Platelet count (10^9^/l) (median (IQR))	164 (95–287)	189 (99–319)	.20
PT (s) (median (IQR))	14.5 (13.3–16.9)	14.5 (13.5–18.1)	.54
APTT (s) (median (IQR))	32.0 (28.3–38.5)	30.0 (26.0–36.0)	.29
Fibrinogen (g/L) (median (IQR))	3.6 (2.7–5.6)	3.8 (2.5–5.4)	.69

ECLS = extracorporeal life support system, P2Y12-inhibitor = P2Y12-adenosinediphosphate-receptor inhibitor, DOAC = direct oral anticoagulant. IQR = interquartile range. Not all laboratory parameters were available in all CRRT circuits; CVVH/RCA2.2 group: hemoglobin 242, platelet count 242, PT 60, APTT 62, and fibrinogen 71 out of 257 circuits and CVVHDF/RCA3.0 group: hemoglobin 257, platelet count 256, PT 68, APTT 65, and fibrinogen 67 out of 290 circuits.

#### CRRT circuit survival

**Figure 2:**
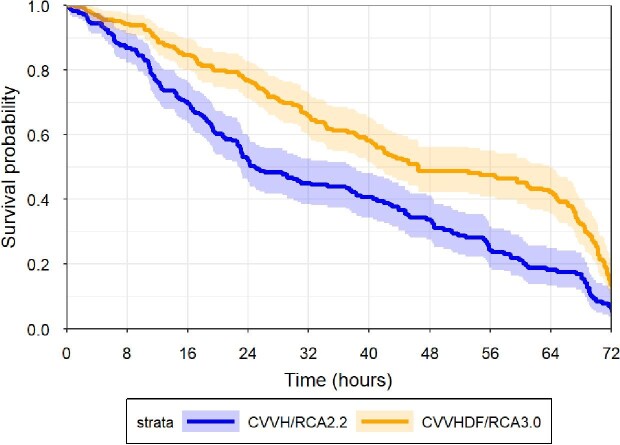
CRRT circuit survival. Kaplan Meijer based on all CRRT circuits of the CVVH/RCA2.2 protocol group (*n* = 179) and the CVVHDF/RCA3.0 protocol group (*n* = 168), after exclusion of CRRT circuits ended because of machine errors, procedures outside the ICU or the decision to cease CRRT. Hazard ratio for circuit failure between groups was 0.31 (CI: 0.18–0.52); *P *< .001.

The median circuit survival was 22.9 (IQR 11.4–48.6) and 39.6 (IQR 19.5–67.3) hours in the CVVH/RCA2.2 and in the CVVHDF/RCA3.0 groups, respectively (*P *< .001) (Table 4). In a frailty Cox proportional hazard model, corrected for sex, body weight, age, APACHE IV score, and COVID-19 status, the hazard ratio for circuit failure was significantly lower in the CVVHDF/RCA3.0 group compared with the CVVH/RCA2.2 group (HR: 0.33 (95% CI: 0.20–0.55); *P *< .001) (Fig. 2, Table 5).

**Table 4:** CRRT circuit survival.

CRRT circuit survival	CVVH/RCA2.2	CVVHDF/RCA3.0	*P* value
**Primary outcome**			
CRRT circuit survival—excluding circuits terminated because of (i) diagnostic procedures or interventions (operations) outside the ICU, (ii) technical errors, and (iii) circuits ended because of the decision to cease CRRT (hours) (median (IQR))	22.9 (11.4–48.6) (*n* = 179)	39.6 (19.5–67.3) (*n* = 168)	<.001
Circuit survival in COVID-19 negative patients (hours) (median (IQR))	22.2 (11.2–44.9) (*n* = 140)	40.8 (23.1–68.4) (*n* = 96)	<.001
Circuit survival in COVID-19 positive patients (hours) (median (IQR))	24.9 (12.0–62.4) (*n* = 39)	36.6 (17.3–66.9) (*n* = 72)	.30
CRRT circuit survival including all circuits			
(hours) (median (IQR))	23.1 (11.8–46.2) (*n* = 257)	32.1 (15.2–65.7) (*n* = 290)	.001

*n* = number of circuits.

**Table 5:** Frailty Cox proportional hazard model CRRT circuit survival.

Model	Hazard ratio (CI)	*P* value
Model 1: Crude CVVHDF/RCA3.0 vs CVVH/RCA2.2	0.31 (0.18–0.52)	<.001
Model 2: adjusted for sex and age	0.31 (0.18–0.52)	<.001
Model 3: adjusted for sex, body weight, and age	0.32 (0.19–0.54)	<.001
Model 4: adjusted for sex, body weight, age, and COVID-19	0.32 (0.19–0.54)	<.001
Model 5: adjusted for sex, body weight, age, COVID-19, and APACHE IV score.	0.33 (0.20–0.55)	<.001

Frailty Cox proportional hazard models based on all CRRT circuits of the CVVH/RCA2.2 protocol group (*n* = 179) and the CVVHDF/RCA3.0 protocol group (*n* = 168), after exclusion of CRRT circuits ended because of procedures outside the ICU, machine errors, or the decision to cease CRRT. Body weight was entered per 10 kg increase.

#### Effect of body weight and convective flow on CRRT circuit survival

**Figure 3:**
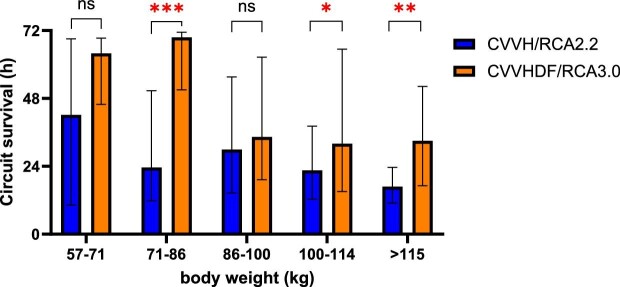
Circuit survival: effect of body weight. Median circuit survival (with interquartile range) per protocolar weight group. Median CRRT circuit survival declined with increasing body weight. Overall circuit survival (both protocol groups together) was significantly lower in body weight categories above 100 kg compared to the body weight category 71–86 kg. Median CRRT circuit survival was significantly higher in the CVVHDF/RCA3.0 protocol group compared to the CVVH/RCA2.2 protocol group in body weight categories 71–86 kg (*P *= 0.00003), 100–114 kg (*P *= 0.03), and >115 kg (*P *= .004).

**Figure 4:**
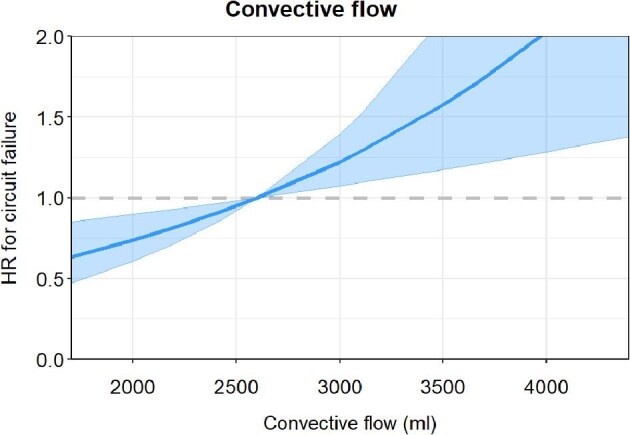
Effect of convective flow on circuit survival. Graphical representation of the association between convective flow and the risk of CRRT circuit failure taking the CRRT circuits from both protocol groups together (*n* = 347), after exclusion of CRRT circuits ended because of machine errors, procedures outside the ICU or the decision to cease CRRT. The blue line shows the hazard ratio for circuit failure. The shaded area correspondents to the 95% pointwise confidence interval.

Median CRRT circuit survival decreased with increasing body weight in both the CVVH/RCA2.2 and CVVHDF/RCA3.0 protocol group (Fig. 3). Within the protocolar body weight groups, as described in Appendix A (Tables S1 and S2), no correlation was found between body weight and circuit survival (Figures S3, Appendix B). The hazard ratio of CRRT circuit failure also increased significantly with increasing convective flow in an overall analysis including both groups (hazard ratio per increase of 100 ml convective flow: 1.05 (95% CI: 1.02–1.09); *P *< .001) (Fig. 4).

#### Prescribed versus delivered CRRT dose and downtime

In patients who received complete per protocol CRRT treatment, the prescribed CRRT dose was 36.9 and 34.9 ml/kg/hour in the CVVH/RCA2.2 (*n* = 39) and CVVHDF/RCA3.0 group (*n* = 52), respectively (*P *= .03) The delivered CRRT doses as percentages of the prescribed CRRT dose during the complete CRRT treatment were 97% and 95% in the CVVH/RCA2.2 and CVVHDF/RCA3.0 groups, respectively (*P *= .54) (Table S7, Appendix B).

#### Electrolyte and metabolic control during CRRT

**Figure 5:**
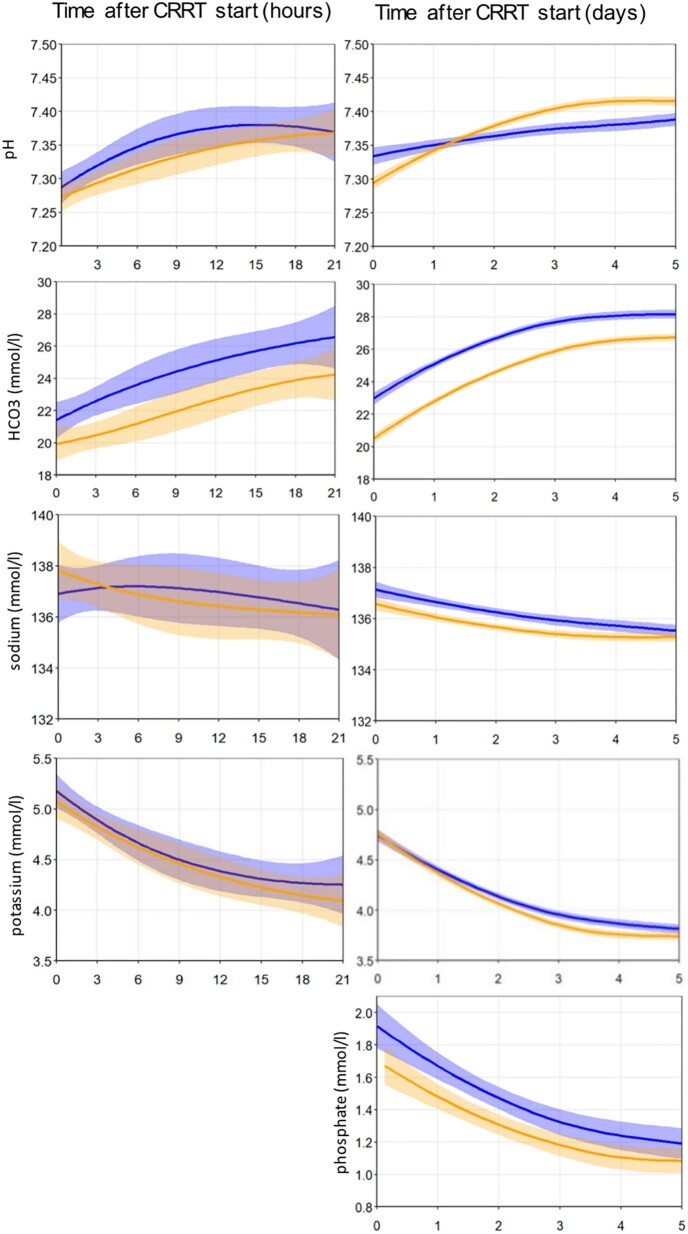
Pattern of electrolytes and acid/base balance during the first 5 days of full course per protocol treatment. Figures are based on data of patients with a complete per protocol treatment in CVVH/RCA2.2 (39 patients, blue line) and in CVVHDF/RCA3.0 (52 patients, orange line). Left panel: the first 21 hours after start of CRRT. Right panel: the first 5 days after start of CRRT.

**Figure 6:**
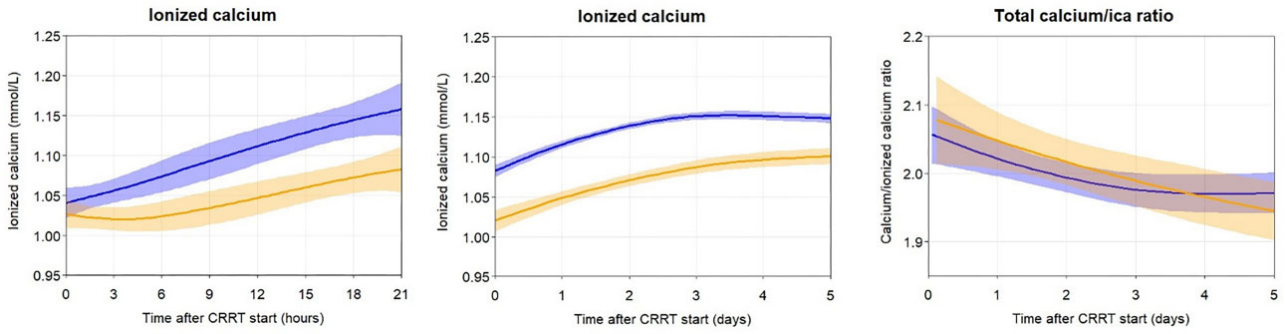
Courses of parameters of citrate metabolism. Figures are based on data of patients with a full course per protocol treatment (39 patients CVVH/RCA2.2; 52 patients CVVHDF/RCA3.0). Left panel: the first 21 hours after start of CRRT, Middle panel: the first 5 days after start of CRRT, Right panel: the first 5 days after start of CRRT.

The rates of metabolic correction of potassium, phosphate, pH, and HCO_3_^−^ were similar in both groups (Fig. 5). Clinically relevant metabolic alkalosis (pH > 7.45 and HCO_3_^−^ >28 mmol/l) occurred in 46% and 19% of patients in the CVVH/RCA2.2 and the CVVHDF/RCA3.0 groups, respectively (*P *= .006). A total calcium/iCa ratio >2.5 at any time during CRRT treatment occurred in 0% and 3.8% of patients in the CVVH/RCA2.2 and CVVHDF/RCA3.0 group, respectively (*P *= .22) (Table 6). The course of ionized calcium was similar in both groups (Fig. 6).

**Table 6:** Citrate accumulation and calcium suppletion during first 5 days of full course treatment.

	CVVH/RCA2.2	CVVHDF/RCA3.0	*P* value
	39 patients	52 patients	
Increased total calcium/ionized calcium ratio (>2.5)			
Patients	0 (0%)	2 (3.8%)	.22
Mean duration per patient (days)	0	1	
Non-lactate high anion gap metabolic acidosis (>12 mmol/l)			
Patients	6 (15.4%)	11 (21.2%)	.48
Mean duration per patient (days)	1.5	1.1	
Calcium-magnesium-chloride solution^a^ replacement rate (ml/min) (median (IQR))	0.11 (0.09–0.12)	0.17 (0.15–0.17)	<.001

^a^Composition of calcium-magnesium-chloride solution: calcium 540 mmol/l, magnesium 240 mmol/l, chloride 1560 mmol/l.

#### Sensitivity analyses

The median circuit survival in patients without COVID-19 was significantly longer in the CVVHDF/RCA3.0 group compared with the CVVH/RCA2.2 group [40.8 (IQR 23.1–68.4) versus 22.2 (IQR 11.2–44.9) hours, respectively; *P *< .001] (Table 4). The median circuit survival in patients with COVID-19, was not significantly different between both groups [36.6 (IQR 17.3–66.9) for CVVHDF/RCA3.0 and 24.9 (IQR 12.0–62.4) for CVVH/RCA2.2 (*P *= .30)] (Table 4). The median CRRT circuit survival in patients treated with a therapeutic dose of LMWH (in 27% and 23% of CRRT circuits in the CVVH/RCA2.2 and CVVHDF/RCA3.0 groups, respectively; *P *= .29) was 38.2 hours (IQR 14.4–58.7) in the CVVH/RCA2.2 group and 62.9 (IQR 24.8–69.0) hours in the CVVHDF/RCA3.0 group (*P *= .03) [Table S6 (Appendix B)]. The median survival of the first CRRT circuit was 33.3 (IQR 13.9–56.6) and 60.3 (IQR 22.1–69.5) hours in the CVVH/RCA2.2 and the CVVHDF/RCA3.0 groups, respectively (*P *= .06) [Table S6 (Appendix B)]. The median circuit survival including all CRRT circuits (thus without exclusion of CRRT circuits that were ended because of machine errors, procedures outside the ICU or the decision to cease CRRT) was 23.1 (IQR 11.8–46.2) and 32.1 (IQR 15.2–65.7) in the CVVH/RCA2.2 and in the CVVHDF/RCA3.0 group, respectively (*P *= .001).

Metabolic alkalosis according to a more sensitive definition (pH > 7.38 and HCO_3_^−^ >26 mmol/l) occurred in 69% and 60% of patients in the CVVH/RCA2.2 and the CVVHDF/RCA3.0 group, respectively (*P *= .35). An increased total calcium/iCa ratio according to a more sensitive definition (>2.25), as applied in our institution, at any time during CRRT treatment occurred in 12.8% and 11.5% of patients in the CVVH/RCA2.2 and CVVHDF/RCA3.0 groups, respectively (*P *= .85).

#### Patient and renal outcomes

Patient and renal outcomes did not significantly differ between both groups (Table 7).

**Table 7:** Patient and renal outcomes.

	CVVH/RCA2.2	CVVHDF/RCA3.0	*P* value
**All included patients**	*n* = 56	*n* = 66	
	257 circuits	290 circuits	
Length of ICU stay (days) (median (IQR))	13.7 (6.6–20.7)	10.7 (5.2–18.8)	.33
ICU mortality	20/56 (35.7%)	26/66 (39.4%)	.68
Hospital mortality	23/56 (41.1%)	28/66 (42.4%)	.88
Reasons for final cessation of CRRT			
Improvement of renal function	14 (25%)	19 (28.8%)	.64
Switch to hemodialysis	23 (41.1%)	17 (25.8%)	.07
Palliative treatment	10 (17.9%)	17 (25.8%)	.29
**Full course per protocol treatment**	*n* = 39	*n* = 52	
	146 circuits	197 circuits	
CRRT treatment duration (days) (median (IQR))	3.6 (2.2–6.0)	4.4 (1.4–9.8)	.84
CRRT circuits per treatment day (median (IQR))	0.6 (0.5–0.9)	0.6 (0.4–0.8)	.37
CRRT circuits per patient (median (IQR))	3 (2–6)	2 (1–5)	.62

## DISCUSSION

The main findings of this study are that circuit survival time was significantly longer in patients treated with the CVVHDF/RCA3.0 protocol compared with those treated with the CVVH/RCA2.2 protocol. In addition, both a higher body weight and higher convective flow were associated with lower circuit survival time.

Notably, the CVVHDF/RCA3.0 protocol differs in three aspects from the CVVH/RCA2.2 protocol: [1] the convective flow was lowered albeit with a near-identical total clearance [2], the citrate dose was increased, and [3] also in non-COVID-19 patients CRRT circuits were primed with 10.000 IU heparin. Therefore, we cannot determine which of the three factors is responsible for the increase in circuit survival. Possibly the lower convective flow in the CVVHDF/RCA3.0 group is the dominant factor explaining the increase in circuit survival time. This suggestion is based on the negative association between the convective flow and CRRT circuit survival time, which is probably a consequence of the lower filtration fraction with CVVHDF treatment. Additionally, higher body weight (that determines the individual convective flow) resulted in decreased CRRT circuit survival. Although, we cannot prove that the convective flow is responsible for the relationship between body weight and circuit survival, this is plausible, since within protocolar body weight groups (having the same convective flow and a different weight within the specific weight group) there was no association between body weight and circuit survival [Appendix A (Tables S1 and S2)]. CRRT circuit survival with CVVHDF was also significantly higher compared to CVVH in a small Chinese randomized controlled trial [8] and in a *post hoc* multivariate analyses in the RICH trial [9]. In another small randomized crossover study, circuit survival was also significantly longer during CVVHDF compared with CVVH, but the prescribed CRRT dose was lower in the CVVHDF group [10]. Concerning citrate dose, in a 2006 study CRRT circuit survival was compared between two patient groups with the same CRRT protocol except for a difference in the citrate concentration in the prefilter substitution fluid (23 mmol/l versus 18 mmol/l). In this study, a higher citrate dose did not increase CRRT circuit survival time, but did result in a higher incidence of metabolic alkalosis defined as pH ≥ 7.50 (75% compared to 28% of patients; *P *= .001) [11]. In another CRRT study comparing RCA doses of 2.5 and 3.0 mmol/l also no differences in CRRT circuit survival were found [12]. However, adjustments in citrate doses based on post-filter iCa levels resulted in too little contrast in the citrate dose between the groups.

Incidences of citrate accumulation were low in both treatment groups. The use of Biphozyl (HCO_3_^−^ 22 mmol/l) instead of Phoxilium (HCO_3_^−^ 30 mmol/l) in the CVVHDF/RCA3.0 group allowed us to use a higher citrate dose with even lower incidences of metabolic alkalosis (19% in the CVVHDF/RCA3.0 group and 46% in the CVVH/RCA2.2 group; *P *= .006). Apart from the development of metabolic alkalosis, metabolic control was similar in both protocol groups.

Future studies should investigate whether CRRT circuit survival can indeed be improved by selectively lowering of convective flow with an equivalent increase in dialysate flow to maintain the same total CRRT dose. These future studies should investigate whether in obese patients a lower delivered dose per kg body weight does optimize CRRT circuit patency. A lower dose per kg body weight might be justified, since total body water in obese patients does not increase relative to the increase in body weight. There is currently no evidence-based method to adjust for this effect. In the meantime, the adjustment that was used in the ATN-trial (adjusted body weight = ideal body weight plus 25% of the difference between ideal and actual weight at ICU admission) can likely be applied [13].

The comparison of the CRRT circuit survival between this and other studies is hampered due to differences in patient selection and a large variation in criteria for selection and replacement of CRRT filters. This heterogenicity becomes evident in the individual studies included in a recent meta-analyses on CRRT survival [14], where three studies included all circuits during treatment [9, 15, 16], while other studies included either only the first circuit [17–21, 22], the first two [23] or four circuits [24], and CRRT circuits were not routinely replaced after 72 hours in three out of 12 studies [16, 22, 23]. Not replacing CRRT filters after 72 hours will lead to higher CRRT circuit survival times. Including only the first, or up to four circuits will also cause overestimation of CRRT survival times. Circuit survival of the first circuit in our study was also higher (Table S6, Appendix B) compared to circuit survival in the primary outcome analysis (Table 4).

Body weight was not mentioned in five studies [16, 17, 19, 23, 24] included in the recent meta-analysis [14], while we found that body weight might be an important determinant of CRRT circuit survival. We decided to include all patients on RCA based CRRT while others excluded patients with a CRRT duration <3 days [19], patients with CKD with prior dependency on dialysis [9, 18, 20], or patients who received a kidney transplant <12 months ago [9].

The strength of our study is the high number of included CRRT circuits in both protocol groups with detailed treatment and laboratory data. Our study has also several limitations due to its retrospective character, due to which some patients received a complete per protocol treatment and others only partial. This, however, reflects clinical practice with a considerable number of patients with liver failure, on extracorporeal life support or with COVID-19 during the study period. We cannot exclude a training effect in study period 2 due to the introduction of the new protocol. We did not routinely measure post-filter iCa levels in the extracorporeal circuit. There is also debate as to whether post-filter iCa levels can be used to assess treatment performance, because analyzers might not reliable assess iCa levels outside the physiological range [25]. Since we included all CRRT circuits with clear exceptions we are confident that our findings do represent the actual performance of both protocols in our tertiary academic mixed ICU patient population.

## CONCLUSION

The median CRRT circuit survival is significantly longer in patients treated with the CVVHDF/RCA3.0 protocol compared with those treated with the CVVH/RCA2.2 protocol.

## Supplementary Material

sfae187_Supplemental_Files

## Data Availability

The anonymized data underlying this article will be shared upon reasonable request to the corresponding author.
