# Supplementary material for: Further improvement of circuit survival in citrate based continuous renal replacement therapy
Source: Clin Kidney J. 2024 Jun 26;17(8):sfae187. doi: 10.1093/ckj/sfae187 (PMC11292220; doi:10.1093/ckj/sfae187)
Supplement: sfae187_Supplemental_Files [file sfae187_supplemental_files.zip › 1263 Revised_Appendix_A_changes_marked (1).docx]

CVVH/RCA2.2 protocol

A summary of the CVVH/RCA2.2 protocol is given in Figure S1. To deliver a mean effluent dose of at least 25 ml/kg/bodyweight/hour over 24 hours, bloodflow varied between 160-220 ml/min, pre-filter Prismocitrate 18/0^®^ replacement flow ranged from 1175-1613 ml/hour and postfilter Phoxillium^®^ replacement flow ranged from 900-2550 ml/hour according to the weight category of the patient (Table S1). Only in patients with COVID-19 the CRRT circuit was primed with 10.000 IU heparin (Leo Pharma BV, Amsterdam, The Netherlands) prior to connection to the patient, except in patients with an increased bleeding risk.

CVVHDF/RCA3.0 protocol

The CVVHDF/RCA3.0 protocol is summarized in Figure S2. To deliver a mean effluent dose of at least 25 ml/kg/bodyweight/hour over 24 hours, bloodflow varied between 150-180 ml/min, pre-filter Regiocit^®^ replacement flow between 1500-1800 ml/hour, post-filter Biphozyl^®^ replacement flow between 100-800 ml/hour and Biphozyl^®^ dialysate flow between 400-1000 ml/hour according to the weight category of the patient (Table S2). Patients with severe hyponatriemia (<125 mmol/l) and (risk of) increased intracranial pressure were excluded from the study. These patients were treated with CVVH with 3.0 mmol/l regional citrate anticoagulation instead of CVVHDF because CVVH is the preferred CRRT mode because it is more iso-osmotic treatment than CVVHDF. In all patients the CRRT circuit was primed with 10.000 IU heparin (Leo Pharma BV, Amsterdam, The Netherlands) prior to connection to the patient, except in patients with an increased bleeding risk.

Composition of replacement fluids and calcium-magnesium substitution.

The type and composition of the replacement fluids is described in Table S3. The composition of Prismocitrate 18/0 is identical to Regiocit, however Prismocitrate 18/0 was replaced by Regiocit by the manufacturer. In the CVVHDF/citrate 3.0 Biphozyl was used instead of Phoxilium, because of both (1) the lower HCO3^-^ content to reduce the risk of development of metabolic alkalosis and (2) Biphozyl contains no calcium, which is a prerequisite for a dialysate fluid, because a calcium containing fluid would undo the effect of citrate administration. In both protocols a calcium-magnesium-chloride solution (calcium 540 mmol/l, magnesium 240 mmol/l, chloride 1560 mmol/l) was administrated via a separate line connected to the accessory infusion port of the central venous catheter (Figure S1 and S2) to compensate for losses. The initial infusion rate of this solution was set at 100%.

In both protocols, the CRRT circuit was routinely replaced after a lifespan of 72 hours.

Laboratory measurements.

In order to detect possible citrate toxicity as a consequence of citrate accumulation (caused by diminished metabolic conversion of citrate to HCO_3_^-^, e.g. in patients with liver failure) resulting in either (1) increased free citrate concentration causing an increased serum anion gap or (2) increased calcium-citrate complex formation resulting in an increased Ca/iCa ratio, iCa, pH, HCO_3_^-^, sodium, potassium, lactate were assessed in addition to daily measurement of total calcium, magnesium and albumin at 1 and 3 hours after the start of CRRT and subsequently every 6 hours during the first treatment day. During the next treatment days these laboratory analyses were performed every 8 hours. In case serum iCa was <1 mmol/l, total calcium was immediately measured for the calculation of the Ca/iCa ratio. Citrate concentration in the extracorporal circuit was lowered to 2.0 mmol/l in the CVVH/RCA2.2 protocol and to 2.5 mmol in the CVVHDF/RCA3.0 protocol if Ca/iCa was >2.25. If Ca/iCa ratio did not fall <2.25, citrate anticoagulation was stopped. If albumin corrected non-lactate high anion gap metabolic acidosis ((Na - Cl - HCO3 - lactate) + 0.25 (40 - serum albumine) >16 mmol/l citrate anticoagulation was stopped. Serum iCa levels were maintained between 0.9 and 1.2 mmol/l by adjusting the calcium-magnesium-chloride solution post-filter flow rate (Table S4).

**Table S1.** Settings of the CVVH/RCA2.2 protocol according to body weight.

| **Body weight** | **Blood flow** | **Convective flow** | | **Total CRRT dose** |
| --- | --- | --- | --- | --- |
| Kg | ml/min | **Prefilter replacement flow**  Prismocitrate^®^  ml/hour | **Postfilter replacement flow**  Phoxilium^®^  ml/hour | ml/hour |
| < 57 | 160 | 1175 | 900 | 2075 |
| 57 - 71 | 170 | 1250 | 1300 | 2550 |
| 71 - 86 | 180 | 1320 | 1700 | 3020 |
| 86 - 100 | 190 | 1395 | 2150 | 3545 |
| 100 - 114 | 200 | 1470 | 2550 | 4020 |
| 115 - 129* | 210 | 1540 | 2550 | 4090 |
| * | 220 | 1613 | 2550 | 4163 |

**Legend to Table S1.** *Bloodflow >200 ml/min can be used when frequent coagulation takes place.

**Table S2.** Settings of the CVVHDF/RCA3.0 protocol according to body weight.

| **Body weight** | **Blood flow** | **Convective flow** | | **Dialysate flow** | **Total CRRT dose** |
| --- | --- | --- | --- | --- | --- |
| Kg | ml/min | **Prefilter replacement flow**  Regiocit^®^  ml/hour | **Postfilter replacement flow**  Biphozyl^®^  ml/hour | Biphozyl^®^  ml/hour | ml/hour |
| < 57 | 150 | 1500 | 100 | 400 | 2000 |
| 57 - 71 | 150 | 1500 | 200 | 800 | 2500 |
| 71 - 86 | 160 | 1600 | 400 | 1000 | 3000 |
| 86 - 100 | 180 | 1800 | 400 | 1000 | 3200 |
| 100 - 114 | 180 | 1800 | 700 | 1000 | 3500 |
| >115 | 180 | 1800 | 800 | 1000 | 3600 |

**Table S3.** Composition of CRRT fluids (all units are in mmol/l).

| Protocol | CVVH/RCA2.2 | | CVVHDF/RCA3.0 | |
| --- | --- | --- | --- | --- |
| Replacement fluid | Prismocitrate 18/0^®^ | Phoxilium^®^ | Regiocit^®^ | Biphozyl® |
| Composition (mmol/l) | Sodium 140  Chloride 86  Potassium 0 Citrate 18 | Sodium 140  Chloride 115,9  Potassium 4  HCO3^-^ 30  Calcium 1.25  Magnesium 0.6  Phosphate 1.2 | Sodium140  Chloride 86  Potassium 0  Citrate 18 | Sodium 140  Chloride 122  Potassium 4  HCO3^-^ 22  Calcium 0  Magnesium 0,75  Phosphate 1,0 |
| Use | Prefilter replacement | Postfilter replacement | Prefilter replacement | Postfilter replacement & dialysate |

**Table S4.** Adjustment of post filter calcium-magnesium-chloride solution infusion rate.

| Serum ionized Calcium level  (mmol/l) | Adjustment infusion rate compared to current infusion rate. |
| --- | --- |
| - 1. - 1.40 | -15% |
| 1.21 - 1.30 | -10% |
| 0.90 - 1.20 | 0% |
| 0.81 - 0.90 | +10% |
| 0.71 - 0.80 | +15% |
| 0.61 - 0.70 | +20% |

**Figure S1.** Schematic overview of the CVVH/RCA2.2 protocol.


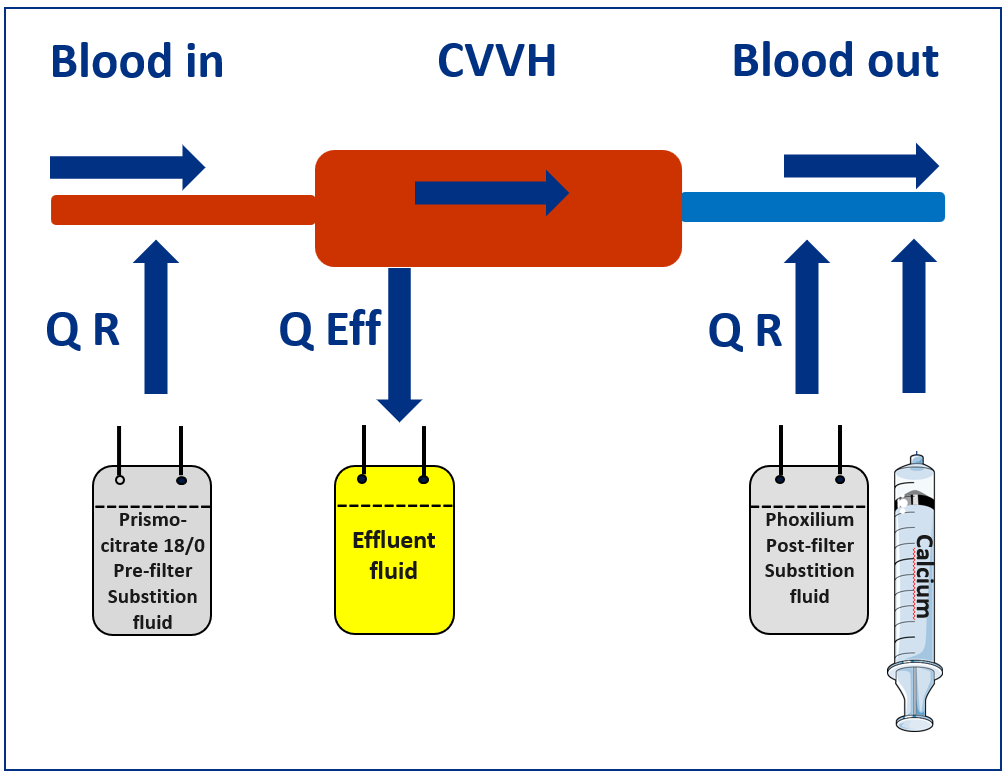


**Legend to Figure S1:** Effluent flow is expressed as Q Eff and determined by convection via filtration in the haemofilter. Q Eff = QR (pre- + post filter) + Q fluid removal. CVVH = continuous veno-venous hemofiltration, QR = replacement fluid rate, Q Eff = effluent fluid rate. Calcium = calcium-magnesium-chloride solution.

**Figure S2.** Schematic overview of the CVVHDF/RCA3.0 protocol.


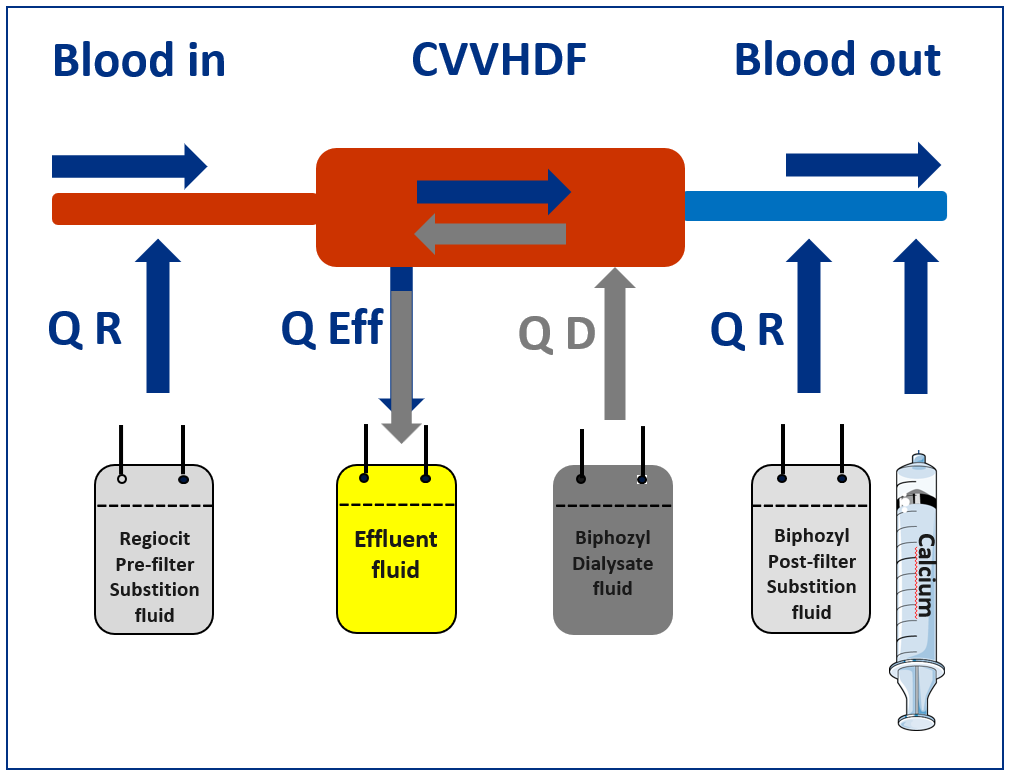


**Legend to Figure S2:** Effluent flow is expressed as Q Eff and is determined by convection via filtration in the haemofilter combined with diffusion via dialysate flow. Q Eff = QR (pre- + post filter) + QD + Q fluid removal. CVVHDF = continuous veno-venous hemodiafiltration QR = replacement fluid rate, Q Eff = effluent fluid rate, QD = dialysate fluid rate. Calcium = calcium-magnesium-chloride solution.
