# Supplementary material for: Further improvement of circuit survival in citrate based continuous renal replacement therapy
Source: Clin Kidney J. 2024 Jun 26;17(8):sfae187. doi: 10.1093/ckj/sfae187 (PMC11292220; doi:10.1093/ckj/sfae187)
Supplement: sfae187_Supplemental_Files [file sfae187_supplemental_files.zip › 1263 Revised_Appendix_B_changes_marked.docx]

**Table S5.** CRRT indications.

|  | CVVH/RCA2.2  n=56 patients | CVVHDF/RCA3.0  n=66 patients | P-value |
| --- | --- | --- | --- |
| Hyperkalaemia | 28 (50%) | 27 (40.9%) | 0.31 |
| Metabolic acidosis | 7 (12.5%) | 13 (19.7%) | 0.28 |
| Fluid overload /anuria /oliguria | 31 (55.4%) | 31 (47.0%) | 0.36 |
| Electrolyte disturbance other than hyperkalaemia | 3 (5.4%) | 2 (3.0%) | 0.66 |
| Uremic complications | 1 (1.8%) | 6 (9.1%) | 0.12 |
| Dependent on dialysis prior to ICU admission | 3 (5.4%) | 5 (7.6%) | 0.62 |
| Renal function decline with need for CRRT according to expert opinion | 2 (3.6%) | 0 (0%) | 0.12 |

**Table S6.** CRRT circuit survival – sensitivity analyses.

| CRRT circuit survival | CVVH/RCA2.2 | CVVHDF/RCA3.0 | P-value |
| --- | --- | --- | --- |
| CRRT circuit survival in patients treated with therapeutic LMWH doses  Excluding circuits ended because of procedures outside the ICU, machine errors or the decision to cease CRRT (hours) (median (IQR))  All circuits (hours) (median (IQR)) | 38.2 (14.4-58.7) (n=49)  38.2 (13.8-57.9) (n=65) | 62.9 (24.8-69.0) (n=38)  42.4 (22.2-68.1) (n=67) | 0.03  0.07 |
| Survival of the first CRRT circuit^1^ |  |  |  |
| Excluding circuits ended because of procedures outside the ICU, machine errors or the decision to cease CRRT (hours) (median (IQR)) | 33.3 (13.9-56.6) (n=40) | 60.3 (22.1-69.5) (n=28) | 0.06 |
| All circuits (hours) (median (IQR)) | 25.1 (12.5-52.0) (n=56) | 40.7 (12.8-67.6) (n=64) | 0.11 |

**Legend to Table S6.** n = number of circuits, LMWH = low molecular weight heparin, ^1^=first day of treatment with regional citrate anticoagulation.

**Table S7.** Prescribed and delivered CRRT dose in patients that received a complete per protocol treatment.

|  | **CVVH/RCA2.2**  39 patients  146 circuits | **CVVHDF/RCA3.0**  52 patients  197circuits | **P-value** |
| --- | --- | --- | --- |
| Prescribed CRRT dose (ml/kg/hour) (median (IQR)) | 36.9 (35.4-39.3) | 34.9 (33.4-37.0) | 0.03 |
| Effectively delivered CRRT dose (ml/kg/hour) (median (IQR)) | 35.8 (31.7-39.5) | 33.0 (31.1-35.8) | 0.06 |
| Delivered dose as percentage of prescribed CRRT dose | 96.7 | 95.3 | 0.54 |

|  | **CVVH/RCA2.2** | **CVVHDF/RCA3.0** | **P-value** |
| --- | --- | --- | --- |
| Blood flow (ml/min) (median (IQR)) | 190.0 (180.0-200.0) | 180.0 (160.0-180.0) | < 0.001 |
| Prefilter substitution flow (ml/kg/hour) (median (IQR)) | 15.0 (13.4-17.4) | 17.8 (16.1-19.8) | < 0.001 |
| Postfilter substitution flow (ml/kg/hour) (median (IQR)) | 21.4 (19.2-23.5) | 4.8 (4.2-6.1) | < 0.001 |
| Dialysate flow rate *(ml/kg/hour) (median (IQR)) | Not applicable | 10.3 (9.0-11.5) |  |

**Table S8:** CRRT settings in all per protocol CRRT circuits.

**Figure S3.** Correlation between circuit survival and body weight within the protocolar body weight groups.

| 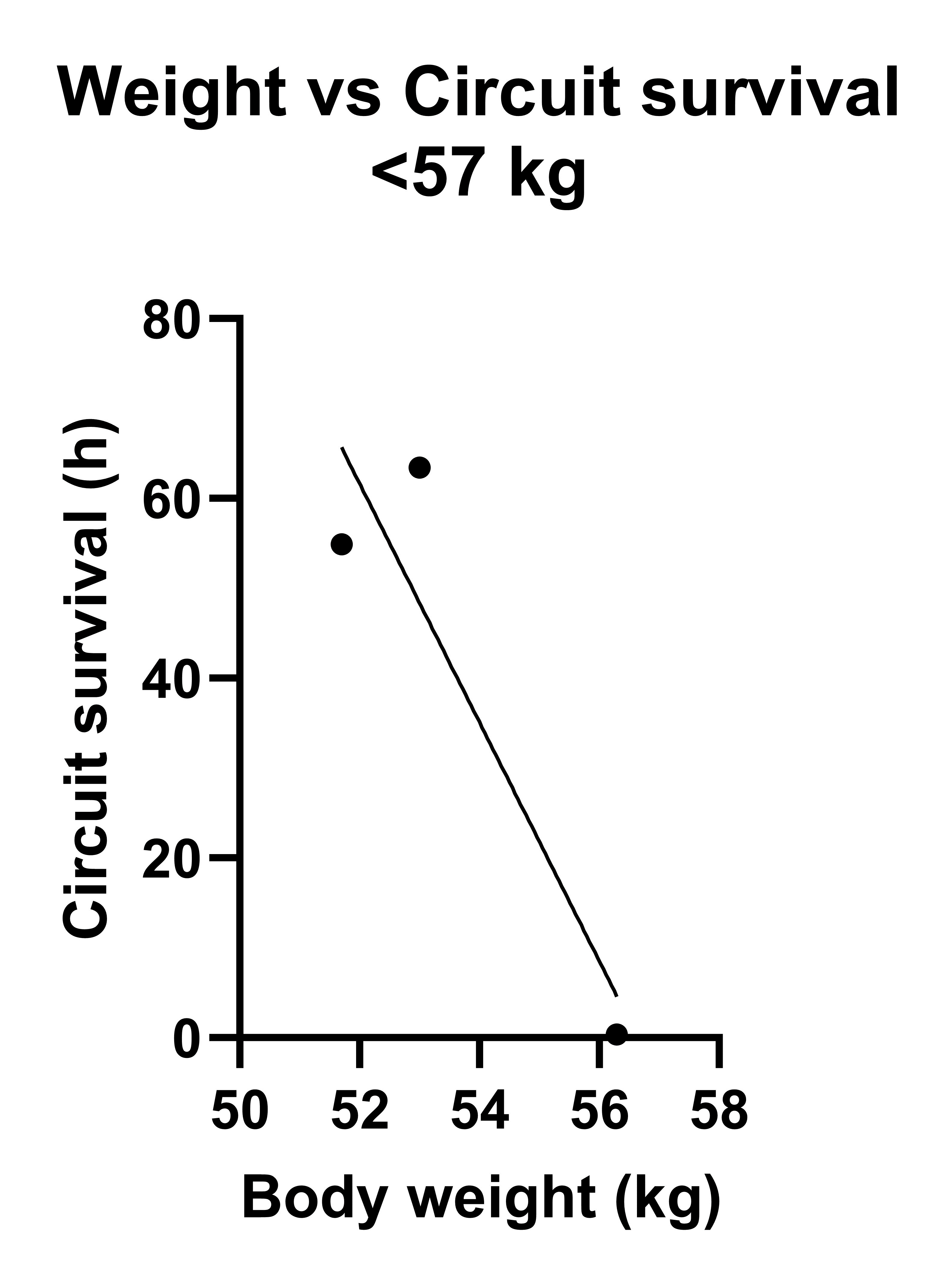 | 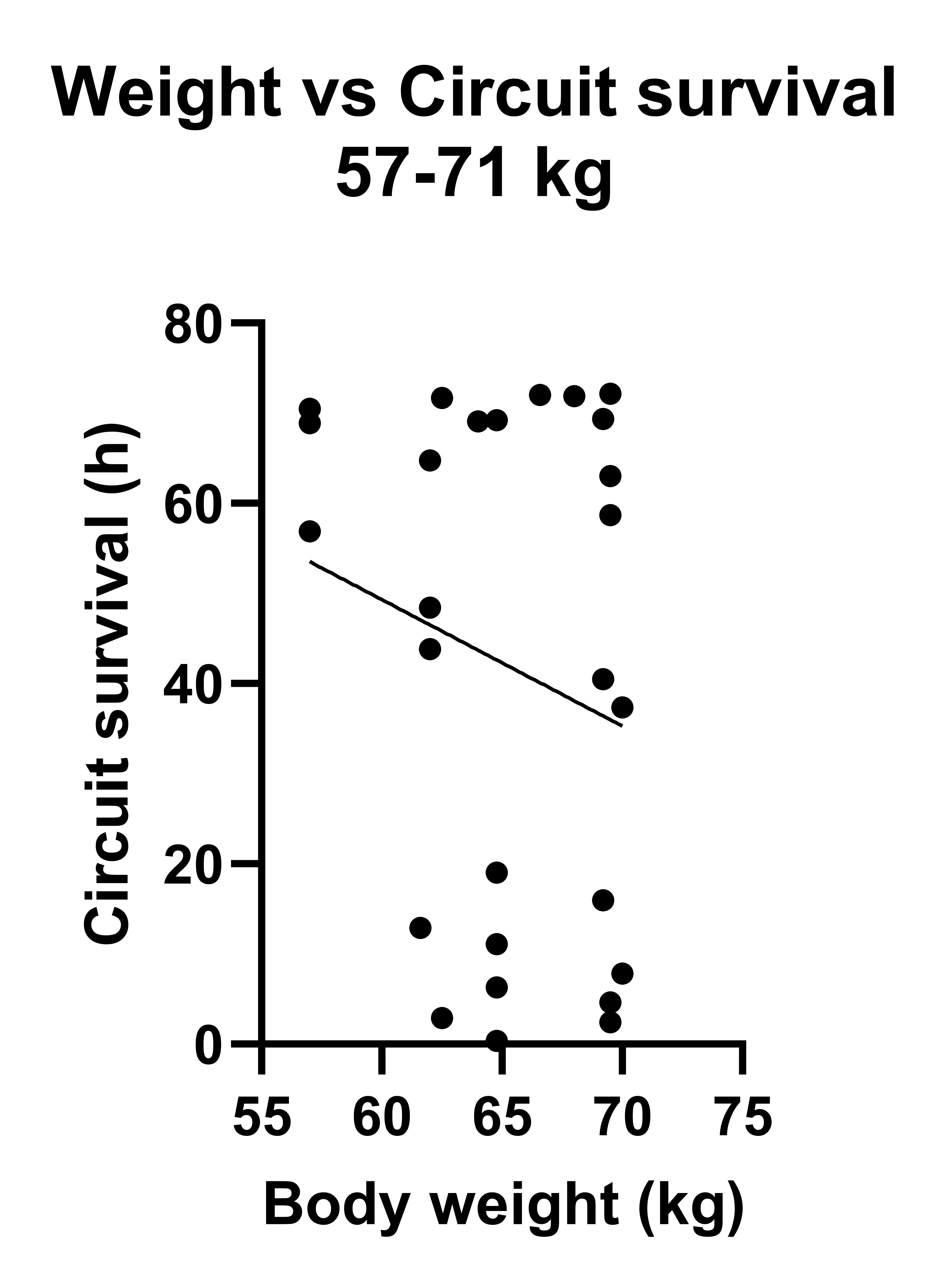 | 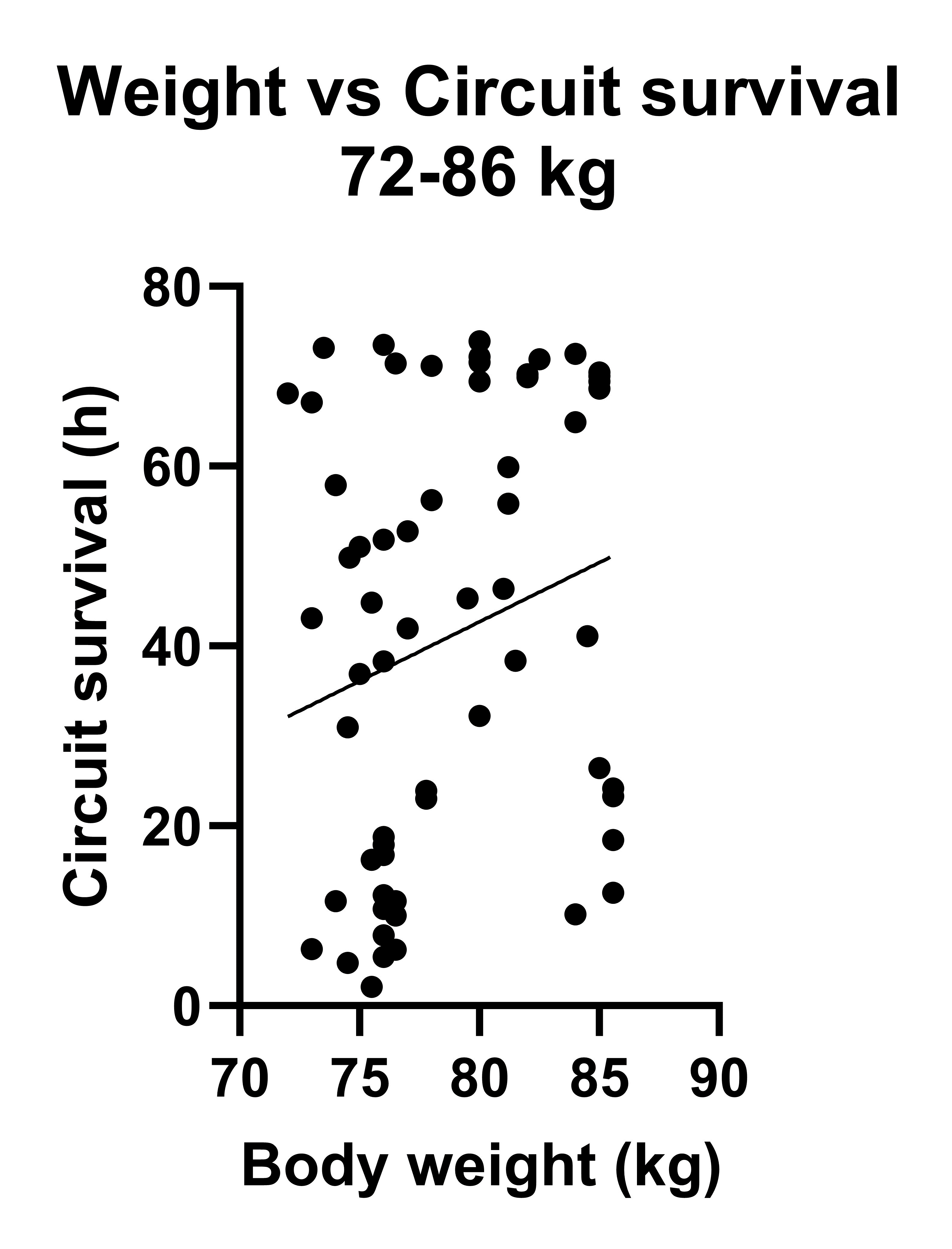 |
| --- | --- | --- |
| 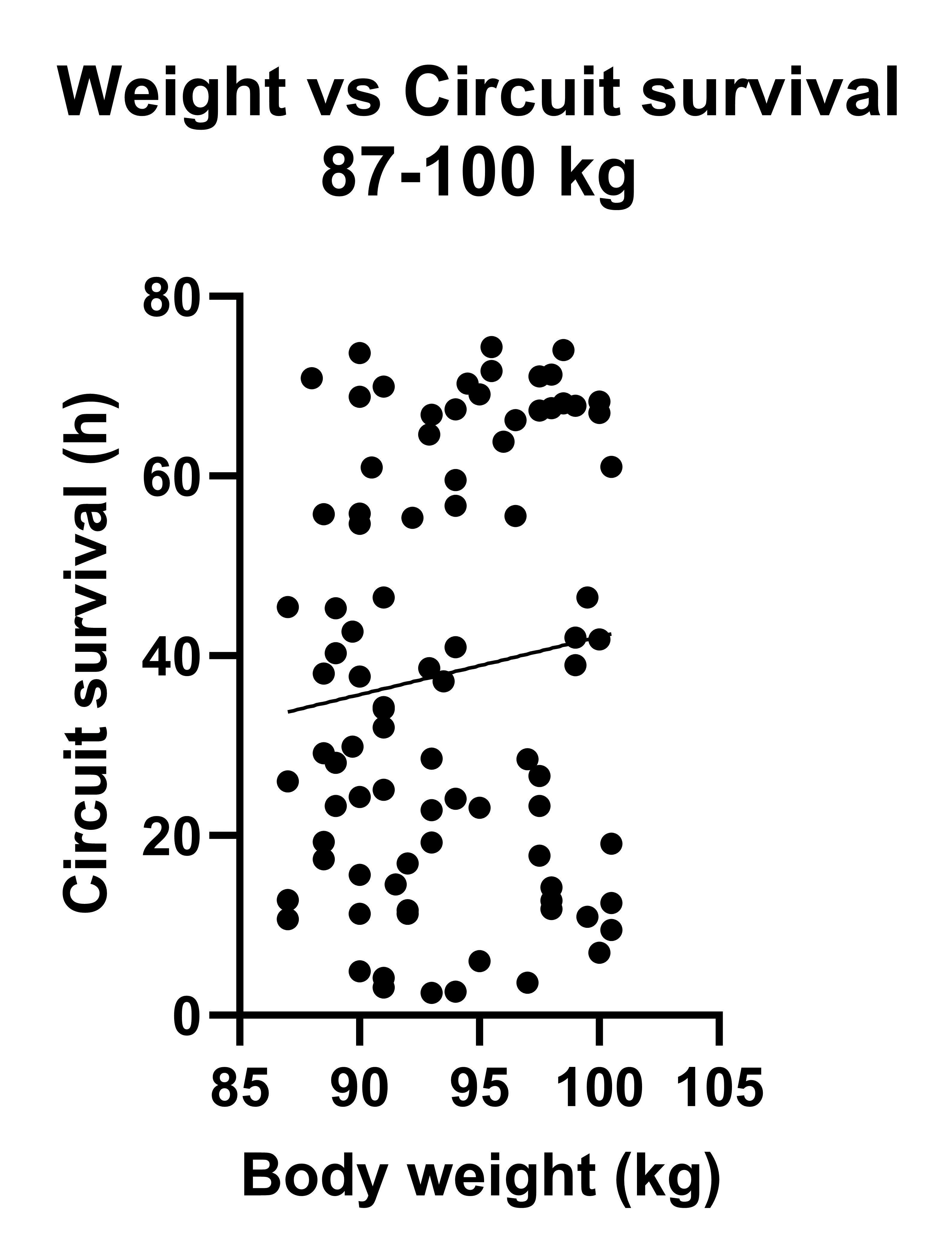 | 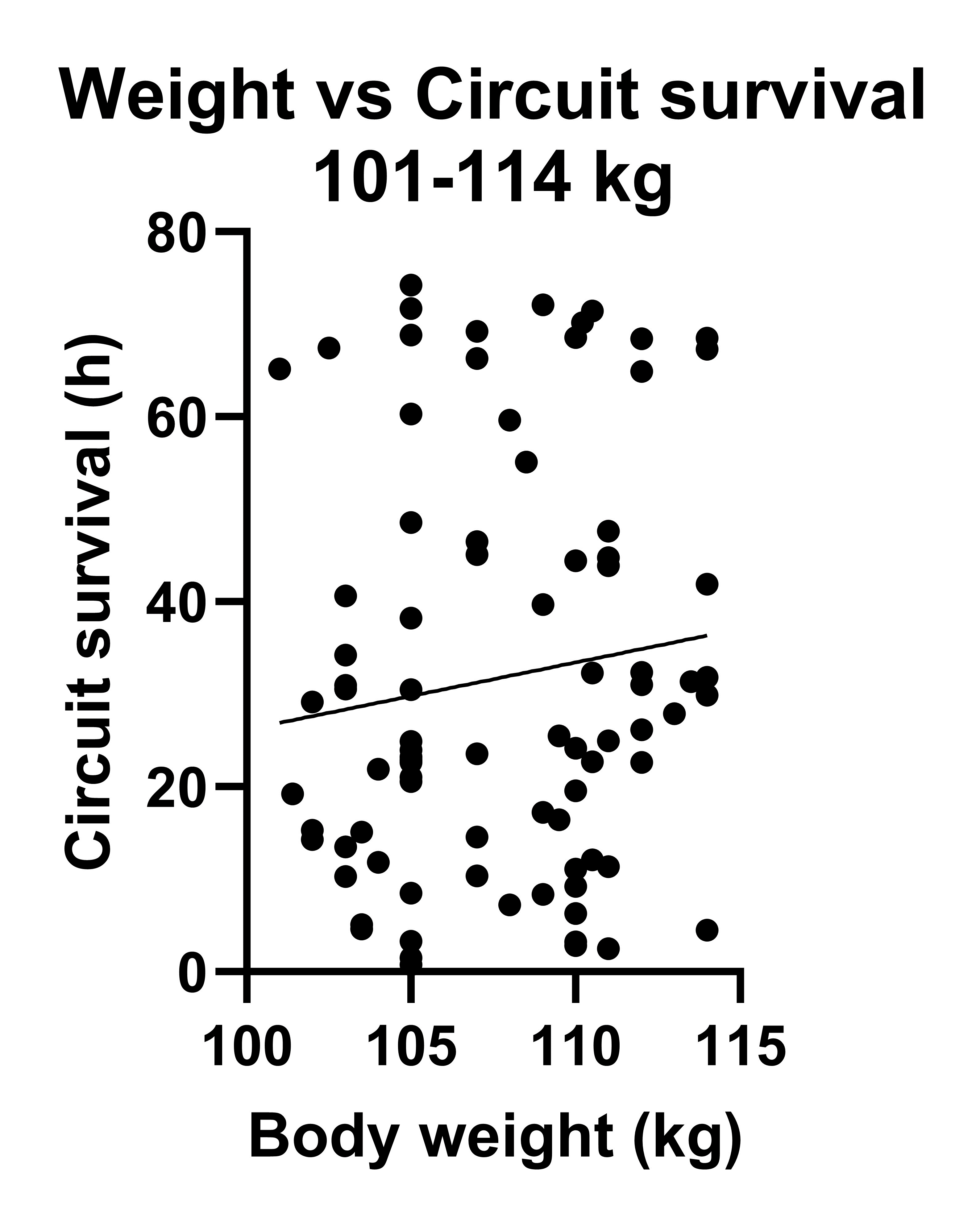 | 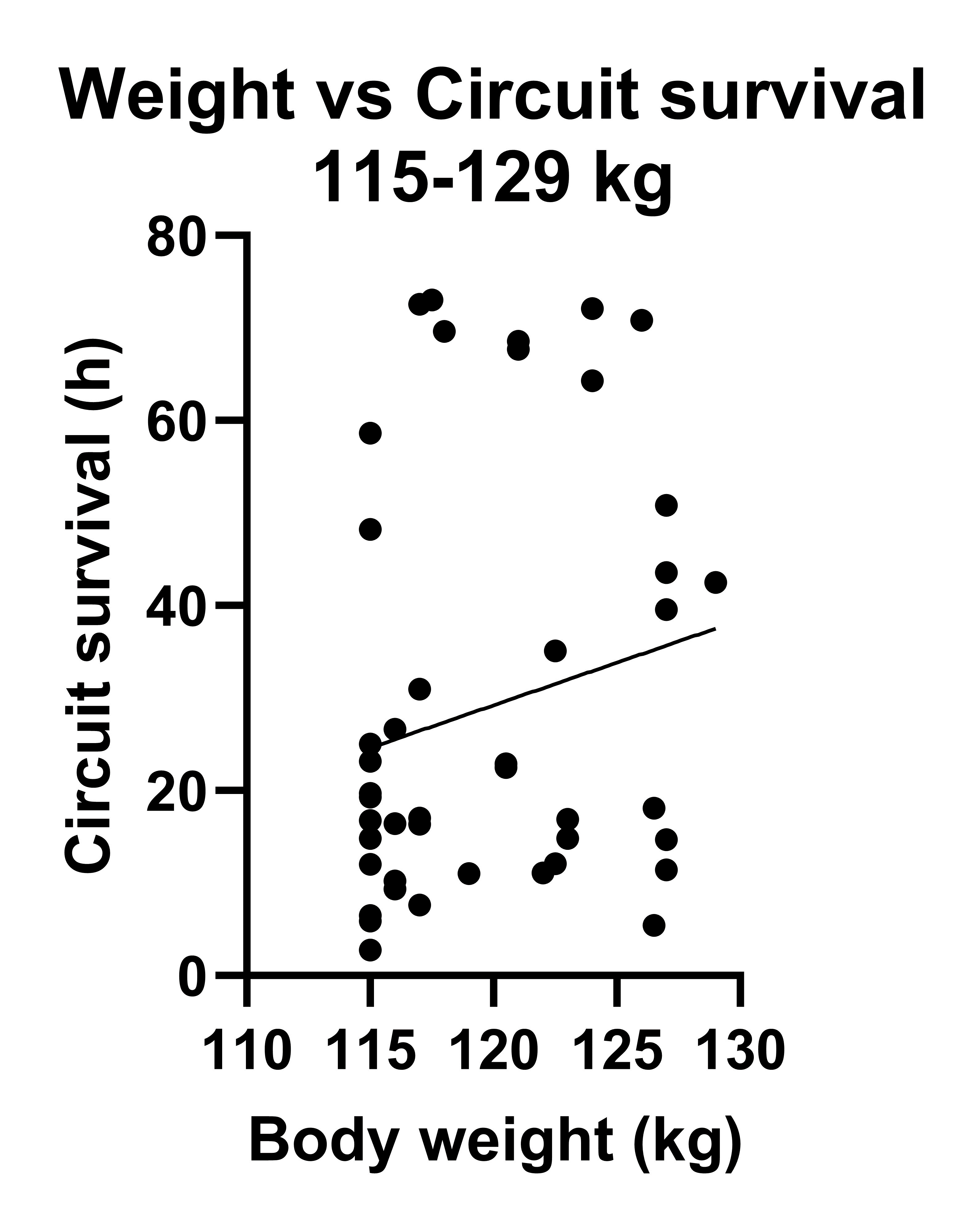 |
| **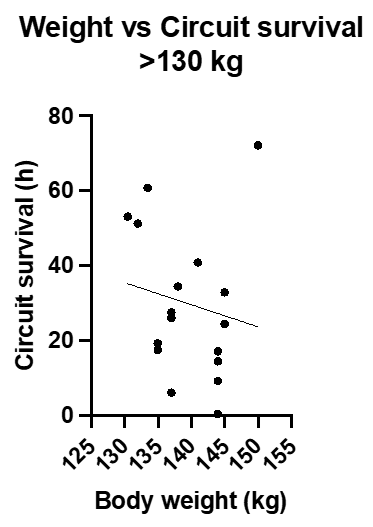** |  |  |

**Legend to Figure S3.** Within each protocolar weight group no significant correlation (Spearman r) was found between body weight and CRRT circuit survival.
